# WHO guideline on the use of non-sugar sweeteners: a need for reconsideration

**DOI:** 10.1038/s41430-023-01314-7

**Published:** 2023-09-18

**Authors:** Tauseef A. Khan, Jennifer J. Lee, Sabrina Ayoub-Charette, Jarvis Clyde Noronha, Nema McGlynn, Laura Chiavaroli, John L. Sievenpiper

**Affiliations:** 1 Department of Nutritional Sciences, Temerty Faculty of Medicine, University of Toronto, Toronto, ON Canada; 2 Toronto 3D Knowledge Synthesis and Clinical Trials Unit, Clinical Nutrition and Risk Factor Modification Centre, St. Michael’s Hospital, Toronto, ON Canada; 3 School of Medicine, Faculty of Medicine, The University of Queensland, Brisbane, QLD Australia; 4 Li Ka Shing Knowledge Institute, St. Michael’s Hospital, Toronto, ON Canada; 5 Department of Medicine, Temerty Faculty of Medicine, University of Toronto, Toronto, ON M5S 1A8 Canada; 6 Division of Endocrinology and Metabolism, Department of Medicine, St. Michael’s Hospital, Toronto, ON Canada

**Keywords:** Risk factors, Nutrition disorders

## Introduction

The World Health Organization’s (WHO) Nutrition and Food Safety Department recently released a guideline on the use of non-sugar sweeteners (NSS) [1] based upon the analysis of a WHO-commissioned systematic review and meta-analysis (SRMA) [2]. The guideline mentions that NSS use in randomized controlled trials (abbreviated as trials) showed a reduction in adiposity outcomes but in prospective cohort studies, NSS intake was associated with increased adiposity and chronic disease risk. Despite conflicting results between the study types, the WHO’s recommendation is very specific: "NSS not be used as a means of achieving weight control or reducing the risk of non-communicable diseases (*conditional recommendation*)”.

We have two major concerns with the WHO guideline, limiting its usefulness, and call for a re-evaluation of the results and recommendation.

## Greater weight given to observational studies

The demonstrated improvement to body weight, BMI, and energy intake outcomes in trials reported by the WHO SRMA are consistent with the results of several other SRMAs of NSS trials that have shown similar benefits for weight loss and BMI [3–7]. In addition, the WHO SRMA also showed that NSS led to reduced sugar and energy intake compared to caloric comparators [2]. These results unequivocally demonstrated that the mechanism of NSS benefit is through a reduction in energy intake. However, results from the prospective cohort studies reported by the WHO SRMA suggested harm with NSS consumption based upon positive associations with BMI, incident obesity, type 2 diabetes, cardiovascular disease, and all-cause and cardiovascular mortality.

In the Grading of Recommendations Assessment, Development and Evaluation (GRADE) approach to rating the certainty of evidence in systematic reviews and meta-analyses, evidence from randomized trials start at high certainty due to its greater protection against bias [8, 9]. Randomization allows confounding factors to be randomly distributed, making it possible to establish a causal relationship between the intervention and the outcome. On the other hand, prospective cohort studies have less protection against bias and cannot establish causality, which is why they start at low certainty in GRADE [8, 9]. When evidence comes from both trials and cohort studies, trials are given precedence [10].

The WHO guideline disregarded the trial evidence and solely relied on the prospective cohort studies, ignoring the established hierarchy of evidence as described by GRADE. The justification for disregarding the trial evidence given was that the results were short-term and thus did not provide evidence of long-term impact. However, this claim is unjustified as the meta-analysis included trials of one-year in duration [11, 12] and some of six months in duration [13–15] with no evidence of effect modification by study duration.

The dismissal of the trial evidence and focus on prospective cohort studies, which are prone to bias and cannot infer causality, is concerning. Such an approach is methodologically flawed as it goes against conventional understanding of nutrition research and best practices in evidence synthesis. In addition, there was no sound biological reasoning provided as to how a consistent benefit on adiposity-related outcomes demonstrated in the trials for up to one-year would develop into a long-term harm.

## Discounting evidence from prospective cohort studies which applied methodologies to reduce bias

Prospective cohort studies follow-up a group of people with an exposure to find out how many reach a certain outcome of interest — this method is referred to as prevalent or baseline analysis [16]. The NSS research community [17–23] and dietary guidelines committees [24, 25] are in agreement that prospective cohort studies using prevalent analysis that investigate NSS’s relationship with cardiometabolic outcomes are at a high risk of bias. This bias is attributed to the high risk of behavior clustering, residual confounding from incomplete adjustment of confounders, and reverse causality (i.e., being at high risk for obesity, type 2 diabetes, and cardiovascular disease leads to increased NSS intake as a risk reduction strategy). The WHO SRMA [2] acknowledged these limitations and presented them as a likely explanation for the negative effect on cardiometabolic outcomes observed in these studies. Despite these limitations, the WHO guideline declared that the harmful associations observed in prospective cohort studies were genuine due to the authors’ efforts to adjust for confounders and reduce bias, even though the authors of the included studies acknowledged the limitations of their own work [26–30].

Prospective cohort studies of NSS using prevalent analysis cannot capture the intended replacement strategy of NSS for excess calories. This results in an underestimate or biased result for the intended cardiometabolic benefit, as evidenced by the contrasting results when compared to the findings from NSS trials. Fortunately, there have been recent advances in analytical methodologies in prospective cohort studies that overcome the limitations of prevalent analyses. These new methods include sequential assessment to measure change in exposure, and substitution analysis modeling NSS as a replacement for caloric sugars. These two robust analytical methods accompanied by adjustment for baseline adiposity substantially reduce the bias associated with NSS studies by capturing the intended substitution of calories, controlling for reverse causation and residual confounding. These rigorous analytical methodologies have now been well-described [18, 20–23] and used in recent published studies [27, 31, 32].

Recently an SRMA of prospective cohort studies of NSS intake was published by Lee at al. that included studies using change analysis of sequential assessments and substitution analysis modeling NSS as a replacement for sugar-sweetened beverages and adjusted for initial adiposity [16]. This SRMA, which included 14 prospective cohort studies with 416,830 participants, showed that an increase in NSS intake (change analysis) in studies with sequential assessments was associated with lower weight and lower waist circumference without any adverse effect on type 2 diabetes. The substitution of NSS beverages for sugar-sweetened beverages was associated with lower weight and lower risk of obesity, coronary heart disease and total and cardiovascular mortality, without any adverse effect on any other cardiometabolic outcomes, including type 2 diabetes. The pooled results from the change and substitution analysis are consistent with the trial evidence on adiposity outcomes [2–7] and support the understanding that NSS intake contributes to weight and cardiometabolic benefits by reducing or displacing excess calories from sugar.

**Fig. 1:**
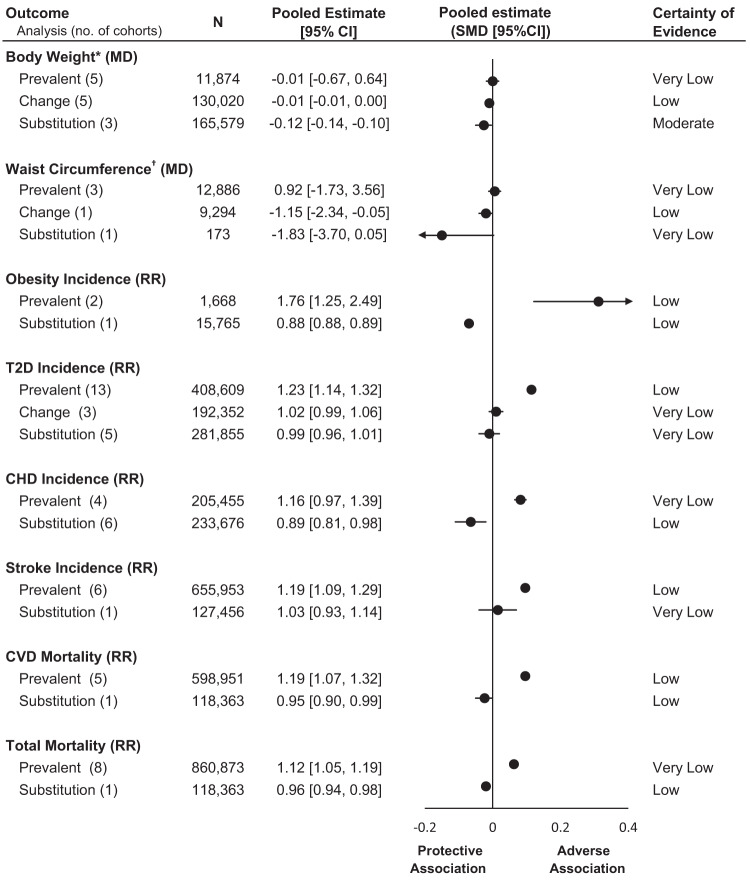
Summary plot of the association between non-nutritive sweeteners (NSS) and cardiometabolic outcomes using prevalent, change, and substitution analysis in cohort studies. Pooled estimates of mean differences (MD) and risk ratios (RR) were converted into standardized mean differences (SMD) to show the estimates among different outcomes on the same scale. Prevalent analyses show the association of NSS and cardiometabolic outcomes and is derived from the WHO SRMA on non-sugar sweeteners [2]. Change analyses show the association between increasing intake of low- and no-calorie sweetened beverages by one serving (330 mL) per day and cardiometabolic outcomes. Substitution analyses show the association between substituting low- and no-calorie sweetened beverages for sugar-sweetened beverages (matched by volume) and cardiometabolic outcomes. Both change and substitution analysis are derived from paper by Lee et al. [16] *Body weight was measured as the mean difference (kg) between high vs. low intake groups for prevalent analysis and as the difference (kg) per year for change and substitution analysis. †Waist circumference was measured as the mean difference (cm) between high vs. low intake groups for prevalent analysis, and as the difference (cm) per year for change and substitution analysis. Abbreviations: CHD coronary heart disease, CVD cardiovascular disease, T2D type 2 diabetes.

Figure 1 summarizes the relationship between NSS and cardiometabolic outcomes using both prevalent (WHO SRMA) [33] and change and substitution analysis (Lee et al.) [16]. The change and substitution analysis shows a neutral or protective association, in contrast to the harmful association shown by the prevalent analysis.

While the WHO guideline acknowledged limitations in the available evidence on NSS’ long-term effects and called out for a better exposure assessment, no effort was made to pool data from studies utilizing rigorous analytical methods. Only one study, the Harvard Pooling Project of Diet and Coronary Disease [34], using a food substitution approach, was cited by the WHO SRMA but the food substitution result was not included in its meta-analysis. This study found a 12% decrease in coronary heart disease risk by replacing sugar-sweetened beverages with NSS beverages. This study was included in the SRMA by Lee et al. al shown in Fig. 1 [16].

We are concerned that the WHO guideline did not consider prospective cohort studies using change and substitution analysis that provided rigorous, biologically plausible, and consistent evidence that mirror those from NSS trials, and instead relied on studies prevalent analysis of NSS that indicated harm. This is a departure from the WHO’s previous approach, as seen in a previous SRMA on saturated and trans fats [35]. The SRMA on saturated and trans fatty acids emphasized the need to carefully consider the impact of nutrient substitution in developing dietary guidelines. In fact, the WHO published an updated report of the effect of substitution of saturated fat and trans-fat intake and with other micronutrients to consider the totality of evidence that is based upon robust methods [36].

## Implications for the WHO guideline

To present a recommendation against the use of NSS for weight control or disease risk reduction — as presented by the WHO guideline — a strong and consistent signal of harm across all study types would be required. However, the available evidence presented by WHO guideline was contradictory, with trials showing benefits for body weight, measures of adiposity and calorie reduction and prospective cohort studies which are susceptible to bias, showing harm for cardiometabolic outcomes. In contrast, a similar assessment of evidence was carried out around the same time by the Diabetes and Nutrition Study Group of the European Association for Study of Diabetes [7, 33], which recommended the use of NSS to replace sugars in beverages and foods as a risk reduction strategy [37].

The WHO guideline also recommends natural sugars from fruit, unsweetened foods and beverages as alternatives for reducing free sugar intake, without conducting any analysis on their effectiveness compared to NSS or providing published data on the subject. It also implied that the diet quality of those who replace free sugars with NSS might may be unaffected. In fact, recent research suggests that NSS users have higher-quality diets and smoke less, but may have a higher prevalence of obesity and type 2 diabetes [38], indicating that NSS consumption may be a response to high disease risk, not a cause of harm [18, 20–23].

## Conclusion

The recommendation of the latest WHO guideline on the use of non-sugar sweeteners relies solely on evidence from long-term prospective cohort studies with prevalent or baseline assessments of NSS without considering change and substitution analysis and ignoring trial data. Prospective cohort studies on this topic using prevalent analysis are subject to serious methodological limitations, and recent evidence from studies with more rigorous analytical methods, modeling change in intake and calorie replacement with NSS, shows benefits for major cardiometabolic outcomes without the evidence of harm. The consistency between trial results and analytically rigorous prospective cohort studies warrants a reconsideration of the WHO’s evidence base and recommendation. In conclusion, both trial and prospective cohort studies, utilizing methods to reduce bias, support the use of NSS in clinical and public health strategies for reducing caloric intake and achieving short and long-term weight loss benefits.

## Data Availability

The datasets generated during and/or analyzed during the current study were from two published reports. The raw data can be extracted from these publications or made available from the corresponding author on reasonable request.
